# Adherence and Blocking of *Candida Albicans* to Cultured Vaginal Epithelial Cells: Treatments to Decrease Adherence

**DOI:** 10.1155/IDOG/2006/98218

**Published:** 2006-07-04

**Authors:** Cara Hollmer, Michael Essmann, Kevin Ault, Bryan Larsen

**Affiliations:** Office of University Research, Des Moines University, 3200 Grand Avenue, Des Moines, IA 50312, USA School of Medicine, Emory University, 69 Jesse Hill Jr Drive, SE Glenn Building, Atlanta, GA 30303, USA

## Abstract

*Background*. Pathogenesis of mucosal microorganisms depends on adherence to the tissues they colonize and infect. For *Candida albicans*, cell surface hydrophobicity may play a significant role in tissue binding ability. *Methods*. A continuous cell line of vaginal epithelial cells (VEC) was grown in keratinocyte serum-free medium (KSFM) with supplements and harvested by trypsinization. VEC were combined with yeast cells to evaluate adherence and inhibition of adherence. In this experimental setup, yeast stained with fluorescein isothiocyanate were allowed to attach to VEC and the resulting fluorescent VEC were detected by flow cytometry. *Results*. VEC were cultured and examined daily after plating and showed morphology similar to basal epithelial cells. Culture media supplemented with estradiol showed increased VEC proliferation initially (first 24 h) but cell morphology was not altered. Fluorescinated *Candida* cells bound effectively to the cultured VEC. Using fresh cells exposed to various preparations of K-Y, we showed that all formulations of the product reduced *Candida* binding to VEC by 25% to 50%. While VEC were generally harvested for use in experiments when they were near confluent growth, we allowed some cultures to grow beyond that point and discovered that cells allowed to become overgrown or stressed appeared to bind yeast cells more effectively. *Conclusion*. Flow cytometry is a useful method for evaluating binding of stained yeast cells to cultured VEC and has demonstrated that commercially available products have the ability to interfere with the process of yeast adherence to epithelial cells.

## INTRODUCTION

The pathogenesis of infections due to *C albicans* and related yeast is a complicated process that may involve a constellation of virulence attributes including fungal morphogenesis, secreted enzymes, biofilm formations, toxin synthesis, and tissue adhesion [1]. In addition, the ability of mucosal pathogens to stably associate on epithelial surfaces has been shown to be important for numerous microbial species that infect mucosal sites. With respect to *C albicans*, even adherence to epithelia may depend on a variety of cellular ligands [2–6]. One feature of fungal surfaces related to tissue binding is cell surface hydrophobicity [7] which we previously explored through attachment of styrene microspheres to yeast [8]. Our earlier studies exploited flow cytometry as a means of evaluating the surface of a large number of yeast cells, predicting on the basis of styrene bead binding, their relative propensity for tissue adherence. These studies, however, were limited in that actual tissue adherence was not measured by the flow cytometry technique. We also found that several marketed intravaginal products could alter the apparent surface hydrophobicity of yeast [9] suggesting that they may be useful as inhibitors of yeast adherence to epithelial cells. A clear limitation of this in vitro was its lack of direct measurement of yeast-epithelial interaction. The ability of commercially available products to inhibit binding of styrene beads to yeast would have more importance if binding to epithelial cells could be demonstrated.

The availability of consistent samples of human vaginal tissue for use in fungal binding studies represents a serious limitation. The use of buccal cells, though readily available, may not be a valid model for vaginal yeast adherence. In addition, the use of exfoliated vaginal or buccal cells with their extant microbial flora would confound assays. Recently, a continuous cell line of vaginal epithelial cells (VEC) [10] has become available and we elected to use these cultured VEC to examine yeast cell binding and explore the possibility that exposure of the VEC to a variety of compounds might mitigate yeast binding.

Previous studies involving yeast attachment to styrene particles [9] showed that several commercially available products could interfere with this process. The same may be true of yeast interaction with vaginal epithelial cells. This study was undertaken to explore that possibility.

## METHODS

The *C albicans* strain used in this research was part of our culture collection and was originally obtained as a clinical isolate from vaginal samples. Yeast strains in our collection are maintained both as frozen stocks for long-term storage and by propagation on Sabouraud's dextrose agar with the intermediate storage at 4°C and subculturing at 3-month intervals. We selected one strain, strain 397, for use in these studies. Prior to use in adherence studies, the test organism was plated on fresh Sabouraud's dextrose agar at 25°C for 48 hours. Previous studies indicated that these growth conditions produced yeast cells that strongly bound styrene beads [8, 9].

Using Robinson et al's work [11] as a guide, fluorescein isothiocyanate (FITC) labeling of yeast cells was carried out according to the protocol of Cantinieaux et al [12] with modifications. Briefly, yeast were harvested from growth media and suspended in carbonate buffer (0.5 M sodium carbonate/sodium bicarbonate, pH 9.5) and cell concentration adjusted to an optical density of 0.74 AU at 620 nm. Fluorescein isothiocyanate (Sigma-Aldrich, St Louis, Mo) was added to make the final concentration of 10 μg/mL and was placed in the dark for 30 minutes at room temperature. Labeled yeast were harvested and suspended in phosphate buffered saline and added to vaginal cells to assess binding as described below.

Vaginal epithelial cells (VEC) that were used in our study have been described elsewhere [10]. Propagation employed keratinocyte serum-free medium (KSFM) obtained from Gibco-Invitrogen (Grand Island, NY) and supplemented with manufacturer-supplied epidermal growth factor and bovine pituitary extract. Growth media also included penicillin (1000 u/mL), streptomycin (1 μg/mL), and Fungizone (0.0125 μg/mL). VEC were grown in 50 mL culture flasks (25 cm^2^ surface area) in 5% CO_2_ and 100% humidity, and refed 3 times weekly until they approached confluence. They were harvested by trypsinization for 5 minutes at room temperature followed by neutralization of the trypsin by addition of Dulbecco's minimal essential median containing 10% fetal bovine serum with gentamicin (25 μg/mL) and vancomycin (250 μg/mL). Harvested cells were either diluted and placed in flasks for continued growth or used in binding studies. VEC used in this study consistently showed greater than 95% viability by trypan blue staining.

For some studies estradiol 17β was added to KSFM. Estradiol 17β (1, 3, 5, [10]-estratriene-3, 17β-diol) was obtained from Sigma-Aldrich and dissolved in methanol as a 1× 10^−6^ M stock solution and kept at −20°C until use. Culture medium supplemented with estradiol contained 5 μL estradiol stock in 5 mL of complete KSFM (1× 10^−9^ M final estradiol concentration).

Flow cytometry was used to demonstrate the yeast binding to VEC. Appropriate conditions for studies were determined experimentally. The VEC displayed autofluorescence that required appropriate adjustment of the instrument. The forward scatter channel was set for logarithmic amplification at a range of 10^−1^ and the side-scatter channel and FL-1h/channel employed logarithmic amplification. Because the assay used an excess of fluorescinated yeast cells (multiplicity of infection = 10), the unbound yeast cells were gated out and VEC events were acquired with the gate turned on, thereby eliminating most of the unbound yeast from analyses. For most analyses, at least 5000 events, presumed to be VEC, were counted.

Inhibitors of yeast binding included several products marketed under the brand name of K-Y (K-Y Jelly, K-Y Liquid. K-Y Ultra, and K-Y Warming) and were donated by manufacturer. K-Y Jelly was diluted 1 : 10 weight per volume prior to use in these studies because of its viscosity. Dilutions were made in phosphate buffered saline.

Data acquired from flow cytometry analysis included a percentage of events that showed fluorescence greater than that due to VEC autofluorescence (percentage of M1) and mean FL1h fluorescence.

## RESULTS

**Figure 1:**
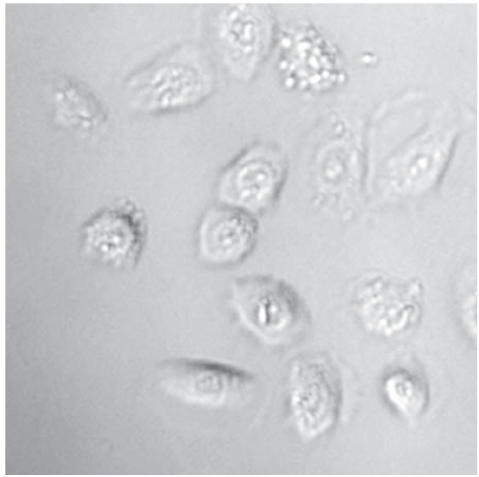
Vaginal epithelial cells cultured at KSFM in polystyrene culture flasks. Photograph taken at ×400 with phase contract microscopy.

VEC were cultured in KSFM medium with supplements as described in the “methods.” These cells grew rapidly and when growth approached confluence provided about 1.2 × 10^6^/50 mL culture flask. Cultured VEC resembled basal cells as seen in Figure 1 and generally did not display fibroblast morphology.

Because relatively few laboratories have worked with these cells, we evaluated the behavior of these cells under a variety of conditions that might relate to in vivo conditions. VEC growth with the addition of estradiol or with the addition of 10% normal human serum was evaluated. Estradiol promoted increased cell proliferation, particularly during the first 24 hours after passage of the cell line. Cell counts in the presence of estrogen were about 25% to 33% greater than in the absence of estrogen at 24 hours but at 48 hours cell counts without estradiol were 1.5 times greater than in cultures with estradiol. Estrogen did not cause any discernible morphologic change to VEC. Growth of VEC with 10% pooled normal human serum did not enhance growth; rather, VEC adhered to culture flasks poorly, cell counts were very low and detached cells aggregated in the medium. As a result of this finding, subsequent experiments employed VEC cultivated without serum and without added estradiol.

**Figure 2:**
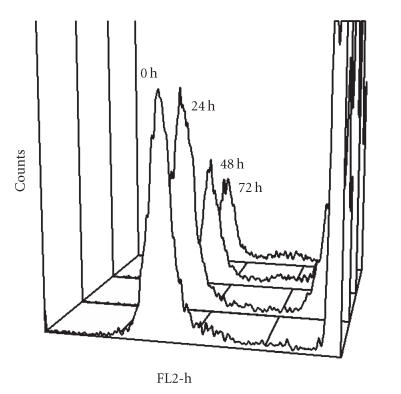
Effect of storage at 4°C on integrity of VEC based on propidium iodide (PI) staining. VEC were harvested and stored in KSFM for 24, 48, and 72 hours and were stained with propidium iodide (0.5 mg/mL in PBS). The *x*-axis shows the relative fluorescence of the particles analyzed. Shift of populations to the right reflects greater PI staining which indicates loss of cell integrity and increasing cell demise. The *y*-axis indicates the number of events (VEC) at each fluorescence level. PI staining yielded a biphasic pattern with the second peak (strong Fl-2 fluorescence) increased from 41.6% among fresh cells to 74.1% at cells stored for 72 hours supporting the use of freshly harvested VEC.

In establishing the parameters for yeast-binding experiments, we determined if VEC, once harvested, could be held overnight to allow additional experiments to be performed on otherwise unused cells. We employed propidium iodide uptake as a measure of cell integrity after harvest and obtained the results shown in Figure 2. As revealed by this study, the proportion of VEC that took up propidium iodide increased daily indicating that under conditions of storage, cell integrity was decreased. From this study, we determined that we would only use freshly harvested cells for this research.

**Figure 3:**
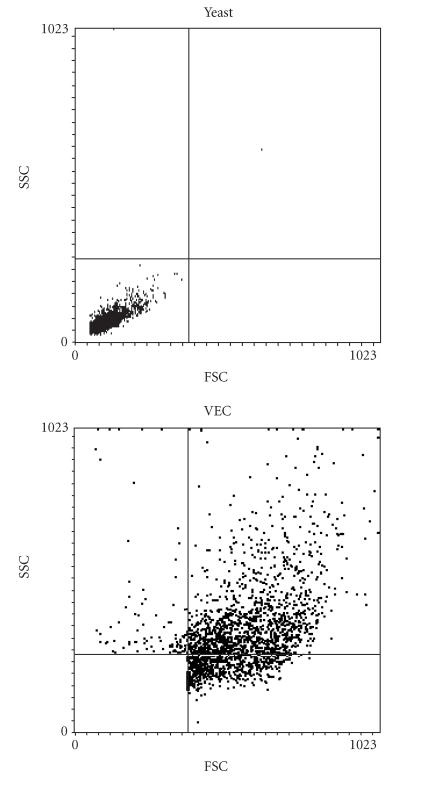
Cytometer setup that permits discrimination of yeast and vaginal epithelial cells (VEC) on the basis of cell geometry. Upper panel shows that 99.7% of yeast cells are segregated into the lower left quadrant. The FSC-SSC distribution of VEC is predominantly in the upper- and right-hand quadrants. We therefore created a gate that includes the upper- and right-hand quadrants in order to analyze the VEC.

Because the ultimate goal of this work was to quantitate yeast binding to VEC using flow cytometry, we established parameters for cytometric analysis experimentally. As shown in Figure 3, VEC had higher forward and side scatter signals than fluorescinated yeast. Yeast cells alone fell into the lower left quadrant of Figure 3, whereas the VEC occupied the other 3 quadrants. Superimposing VEC and yeast plots indicated little overlap between these populations and also suggested that we could create a gate that would exclude virtually all unbound yeast cells. Fluorescence from cells in the upper and right quadrants would be attributed to VEC with fluorescinated yeast (Figure 3).

**Figure 4:**
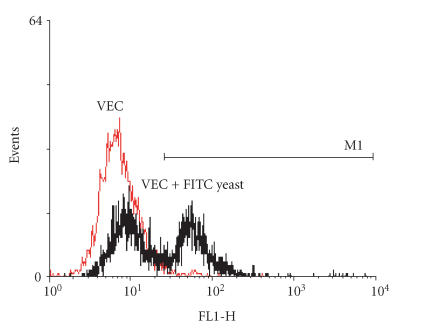
FITC-stained yeast binding to VEC is illustrated. VEC alone have significant autofluorescence as indicated by the gray histogram. When stained, yeast become attached to VEC providing a second fluorescent peak at a level approximately one log greater than VEC autofluorescence.

Having established the flow cytometry parameters to be used in this study, we next determined the ability of fluorescinated yeast to bind to VEC and to contribute to the fluorescence of the VEC. As shown in Figure 4, VEC alone showed a peak of autofluorescence on the FL1h histogram. However, when combined with labeled yeast, two fluorescent peaks were identified, the second peak reflected the fluorescence of the yeast attached to the VEC. Based on this pattern of fluorescence, 90% of VEC appeared to bind yeast.

**Figure 5:**
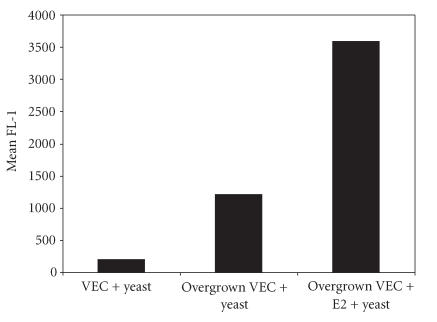
The effect of environmental conditions on yeast adherence to VEC. Controls consisted of VEC incubated with fluorescenced yeast for 30 minutes before analysis. VEC obtained from cultures that had grown beyond the time they would ordinarily be harvested showed greater relative fluorescence than did cells harvested on a normal schedule. VEC undergoing prolonged cultivation in the presence of 1.0 × 10^−9^ estradiol (E2) showed the highest level of fluorescence due to fluorescent yeast adherence.

Because we were interested in the binding of yeast to VEC cultivated under conditions more reflective of in vivo conditions, we examined overgrown cell cultures to determine if they showed different binding characteristics compared to nonconfluent cultures. In vivo, vaginal epithelial cells mature from the metabolically active basal layer to become superficial, pyknotic squames. This process is believed to be related to the superficial layer of the vagina having migrated away from the underlying basement membrane with attendant nutrient depletion and exposure to environmental stress. We postulated that allowing cell cultures to grow beyond the point at which we normally harvest them may produce cells somewhat more like those that exist at the vaginal lumen. Consequently, we determined if growing cells to densities greater than normal would alter binding properties. VEC were allowed to grow two days beyond the time at which we normally harvested them. As shown by data in Figure 5, allowing VEC to become overgrown actually increased the fluorescence due to yeast binding nearly threefold. As indicated in the same figure, addition of estradiol, which caused an initial increase in cell proliferation, also seemed to support increased yeast binding.

**Figure 6:**
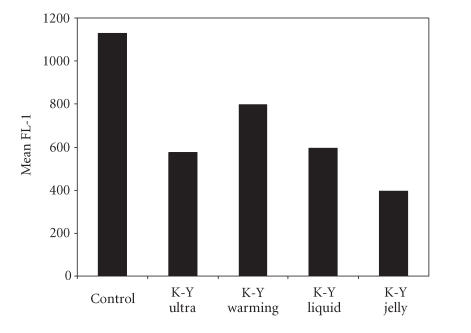
The amount of FL-1 fluorescence indicates the relative density of yeast binding to VEC. Controls represent fluorescinated yeast bound to VEC. The remaining bars indicate the binding of yeast to VEC previously treated with each of 4 K-Y products. The lower fluorescence after K-Y treatment indicates reduced yeast binding to VEC.

Because our prior studies with styrene microspheres indicated that several K-Y products decreased the ability of yeast to bind the hydrophobic beads [9], we determined if these same intravaginal products affected binding of yeast to VEC. As illustrated in Figure 6, each of the products tested altered the binding of yeast as indicated by diminished fluorescence compared to controls. While yeast adherence was decreased under these conditions, it was clearly not eliminated entirely.

## DISCUSSION

Investigation of fungal interactions with epithelium has been methodologically challenging, with various methods being pursued by different authors. One model system employs microscopic evaluation of exfoliated vaginal cells [13, 14] incubated with microorganisms. Buccal cells have been used as a surrogate for vaginal cells although these may not be entirely comparable [15], though they do have the advantage of being relatively more available. Variations on these techniques have included examination of exfoliated cells from various sites [16] in a range of women (eg, pregnant, nonpregnant, diabetic) as reported by Segal et al [17] or adherence of organisms to cells derived from variant epithelial tissues (normal versus cancerous) [18].

To gain more consistency in observations, cultured cells have been used. Cultured HeLa cells, originally derived from cervical cancer, have been employed in controlled adherence studies and can be exposed to yeast cultivated under various conditions [19]. The disadvantage of using HeLa or other similar cells is the possibility of the cells changing over many thousands of passages of culture. Moreover, the fact that HeLa cells were explanted from a neoplasm suggests another intrinsic difference from normal vaginal epithelium. While the use of explanted or exfoliated cells and tissues directly harvested from humans has at least the appearance of being more physiologic, it also has the disadvantage of being more difficult to employ in a controlled experiment because of differences between donor cells. The present study exploited the continuous culture of VEC line that has not been widely available previously.

Our laboratory has been engaged in developing flow cytometry methods for characterizing microorganisms and their interactions with surfaces [8, 9], because the techniques are facile and generate significant amounts of data (typical flow cytometry readings evaluate 5,000–10,000 cells for each sample tested). Appropriate development included establishing the level of background fluorescence exhibited by cultured VEC. Having previously examined exfoliated buccal cells (data not shown) which had a high level of endogenous fluorescence, we were uncertain if VEC would likewise show unacceptably high levels of fluorescence. As shown in this report, we were able to appropriately adjust the cytometer parameters such that unbound fluorescinated yeast cells could be differentiated from unstained vaginal epithelial cells on the basis of particle size and fluorescence and vaginal epithelial cells fluorescently stained by the yeast attached to them.

Our method still suffered from the limitation that the cultured vaginal epithelial cells were more like basal cells than the squamous cells of the vaginal lumen. It is generally believed that the maturation of VEC from basal cells to luminal squames is the result of the migration of these cells away from the basement membrane with accompanying privation of nutrients and crowding during the proliferative process. We attempted to introduce stress into our cell cultures that might mimic epithelial proliferation by allowing them to overgrow and by harvesting them later than we normally would. We found that these presumably stressed cells bound more fluorescinated yeast than did cells harvested on the normal schedule. Viability testing with trypan blue indicated that the cells from overgrown cultures were still viable. This experiment is admittedly an imperfect representation of the cell maturation process that occurs in vivo and should probably be followed up by the development of raft cultures [20]. However, raft cell culture was beyond the scope of the present investigation. Notably, introducing estrogen into this experiment further increased yeast binding to VEC and suggests an additional variable that may be investigated in raft cultures.

A practical result of the work identified several marketed intravaginal products which were able to interfere with the attachment of yeast to VEC under the experimental conditions of this study. While this knowledge is not presumed to provide a new method of treating or preventing fungal vaginitis, it does suggest that components of these marketed products may be useful as vehicles for delivering topical antimycotics and could offer a therapeutic advantage in such a context. Previous research from our laboratory indicated that bioadhesive vehicles containing an active antifungal drug may influence the efficacy of the test drug [21] and suggests that the matrix for delivering topical antifungals deserves more attention in order to improve currently available products.
